# Geometric Deep Lean Learning: Deep Learning in Industry 4.0 Cyber–Physical Complex Networks

**DOI:** 10.3390/s20030763

**Published:** 2020-01-30

**Authors:** Javier Villalba-Díez, Martin Molina, Joaquín Ordieres-Meré, Shengjing Sun, Daniel Schmidt, Wanja Wellbrock

**Affiliations:** 1 Fakultaet fuer Management und Vertrieb, Campus Schwäbisch-Hall, Hochschule Heilbronn, 74523 Schwäbisch-Hall, Germany; wanja.wellbrock@hs-heilbronn.de; 2 Department of Artificial Intelligence, Universidad Politécnica de Madrid, Campus de Montegancedo, 28660 Boadilla del Monte, Madrid, Spain; martin.molina@upm.es; 3 Escuela Técnica Superior de Ingenieros Industriales, Universidad Politécnica de Madrid, José Gutiérrez Abascal 2, 28006 Madrid, Spain; j.ordieres@upm.es (J.O.-M.); shengjing.sun@alumnos.upm.es (S.S.); daniel.schmidt@saueressig.de (D.S.); 4 Exposure, Epidemiology, and Risk Program, Department of Environmental Health, Harvard T.H. Chan School of Public Health, Boston, MA 02115, USA; 5 Lead Developer Quality Inspection, Matthews International GmbH, Gutenbergstraße 1-3, 48691 Vreden, Germany

**Keywords:** Industry 4.0, IIoT, geometric deep learning, lean management

## Abstract

In the near future, value streams associated with Industry 4.0 will be formed by interconnected cyber–physical elements forming complex networks that generate huge amounts of data in real time. The success or failure of industry leaders interested in the continuous improvement of lean management systems in this context is determined by their ability to recognize behavioral patterns in these big data structured within non-Euclidean domains, such as these dynamic sociotechnical complex networks. We assume that artificial intelligence in general and deep learning in particular may be able to help find useful patterns of behavior in 4.0 industrial environments in the lean management of cyber–physical systems. However, although these technologies have meant a paradigm shift in the resolution of complex problems in the past, the traditional methods of deep learning, focused on image or video analysis, both with regular structures, are not able to help in this specific field. This is why this work focuses on proposing geometric deep lean learning, a mathematical methodology that describes deep-lean-learning operations such as convolution and pooling on cyber–physical Industry 4.0 graphs. Geometric deep lean learning is expected to positively support sustainable organizational growth because customers and suppliers ought to be able to reach new levels of transparency and traceability on the quality and efficiency of processes that generate new business for both, hence generating new products, services, and cooperation opportunities in a cyber–physical environment.

## 1. Introduction

Today it seems almost a truism to talk about the fact that data surround us. According to recent studies, by 2025 humanity will have created about 163 zettabytes of information [1]. However, the alarming thing is not that we are going to be flooded with data, but that these data will be very different from the data with which we are used to dealing in classical disciplines such as signal or image processing, statistics, or machine learning. Beyond this, the data we will face are data that will emerge from the trillions of objects connected to the Internet of Things (IoT). In many cases, including the industrial IoT (IIoT), these data are produced by distributed sources, such as thousands of sensors in factories, i.e., data are distributed over networks. Managing large amounts of data in these ever-expanding networks raises nontrivial concerns about the efficiency of data collection, processing, analysis, and security [2,3]. Currently, data from processes and systems are collected and stored without a clear strategy, and this can be a barrier to implementing paradigms such as “social manufacturing” [4]. In addition to being distributed, these data may be unstructured, and therefore cannot generally be encapsulated in one table. A defined strategy is therefore needed on what kind of data to collect at the technical and the organizational level. Finally, in addition to numerical, data can be ordinal, categorical, or other. The aim of this work is to introduce the reader to a series of concepts that pave the way for processing these data by means of adapted deep-learning techniques [5].

The purpose of this work is to study the possibility of providing Industry 4.0 leaders with a theoretical model that allows for the extraction of relevant patterns embedded within their organizations by means of artificial intelligence. Specifically, the goal of this work is to provide the reader with mathematical models that adapt convolutional and pooling deep-learning operations, hence describing the possible use of geometric deep-learning architectures on non-Euclidean Industry 4.0 complex cyber–physical networks. The structure of this work is as follows: First, Section 2 provides relevant background information, clarification, and definitions. Second, Section 3 provides a framework of previous relevant concepts regarding deep learning, specifically geometric deep learning. Third, Section 4 provides mathematical models to compute geometric-deep-learning algorithms over Industry 4.0 lean-management complex-networked cyber–physical systems. Finally, Section 5 outlines the conclusions and managerial implications of this model, and its applications in the field.

## 2. Background

This brief section presents and defines fundamental preliminary concepts to the comprehensive understanding of the presented content in the following sections of this work:

- Industry 4.0. The term Industry 4.0 has gained large traction since it was first publicized [6], stating the need for a paradigm shift towards a less centrally controlled manufacturing structure. It is seen as the Fourth Industrial Revolution, with the first three being mechanization through steam power, mass production through electrically operated engineering, and the digital revolution through the integration of electronics and IT. Industry 4.0 enables more production autonomy as technology becomes more interconnected, and machines are able to influence each other by creating a cyber–physical system.
- Cyber–Physical Systems. The term “cyber–physical system” in the context of Industry 4.0 refers to the tight conjoining of and co-ordination between computational and physical resources. The impact on the development of such systems is a new paradigm of technical systems based on collaborative embedded software systems [7].
- Lean Management. Lean-management systems in an Industry 4.0 cyber–physical context have been described as sociotechnical entities that aim to systematically reduce the variability of value-creation processes [8,9,10,11,12,13]. These two fundamental dimensions, the social and the technical, are subsequently meant to symbiotically support each other to maximize value creation through the systematic elimination of activities that do not add value for the client. A series of models were presented by scholars that allow the analysis and quantification of these systems as complex networks [14,15].
- Complex-Networked Organizational Design. Under the organizational-network paradigm, modern Industry 4.0 cyber–physical lean-management-oriented organizations can be understood as a symbiotic sociotechnical ecosystem of social networks [16] that interacts with increasingly complex-networked physically distributed interconnected sensors [17], whose readings are modeled as time-dependent signals on the vertices, human or cyber–physical, respectively. This means that, on the nodes of the network, attributes can be found that describe them as having the form of a given time series.
  Within this framework, a complex network is defined as a graph with nontrivial topological features that do not occur in simple graphs such as lattices and random networks [18]. For any given time *t*, lean complex cyber–physical networks can be formally described by time-dependent graphs Ω(t)=[N(t);E(t)] that can be understood as lists of N(t) nodes and E(t)⊂(N(t)xN(t)) edges that represent its human and cyber–physical nodes, and its standard communication edges [19]. Given the static graph in *t*, Ω(t), each node and edge can be characterized by a series of typically two-dimensional signals $x=\left[x_{1},\ldots ,x_{n}\right]\in \left(R^{n}xR^{m}\right)$, where *n* relevant parameters of the node or axis are described as the time series of *m* elements. In the case of nodes, signals typically represent demographic, sociological, or competence information. In case that the nodes are human, and in the case of a cyber–physical node, relevant information on the state of the cyber–physical node expressed in time series of several key performance indicators. In the case of edges, signals typically represent information referring to the quality of measurable relationships of the individual with other stakeholders of the organization; in the case of human–human or cyber–physical-to-human edges, of the time series associated with relevant key performance indicators being reported to other stakeholders. Specifically, snapshots for the time-dependent graph can be built, that is, the time-dependent graph is considered as an ordered pair of potentially different sets. A time-dependent graph considered as a sequence of static graphs is given by Expression 1.
  (1) $$\Omega =[\Omega \left(t_{1}\right),\Omega \left(t_{2}\right),\ldots ,\Omega \left(t_{k}\right)]$$
  This method is most commonly used for modeling discrete time-dependent graphs, and is suitable for the time-dependent graph with a specific time structure, especially in real-time networks such as complex-networked cyber–physical systems [20]. This modelling method is assumed here, and the time sequence of static graphs is not explicitly mentioned when referring to time-dependent graphs.

As a consequence of these references, it can be stated that cyber–physical complex-networked lean-management systems in an Industry 4.0 context can be understood as management systems that systematically try to reduce the intrinsic variability of industrial value-creation processes by understanding them as complex networks of computational and physical elements.

## 3. Related Work

**Figure 1:**
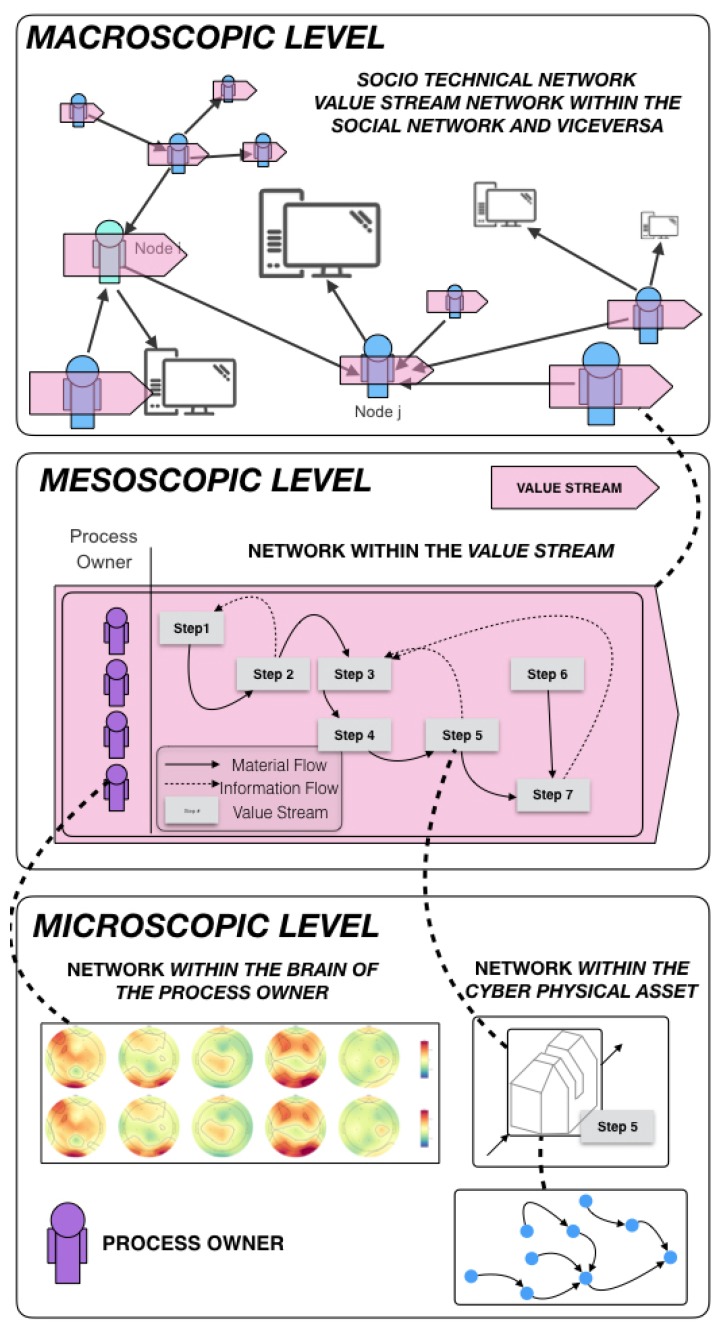
Macroscopic, mesoscopic, and microscopic levels of organizational sociotechnical complexity.

Within this framework, the work approaches the interpretation of strategic information contained in Industry 4.0 cyber–physical complex-networked lean-management systems from two main vectors: social and technical strategic organizational design complexity. As shown in the research overview in Table 1, these two research directions have been intensively examined at three (micro-, meso-, and macroscopic) levels of complexity. A better visualization of these organizational levels is in the graphical abstract of Figure 1 for clarity purposes, but it should be noted that this classification is purely synthetic; in reality, cyber–physical systems in an Industry 4.0 context present the continuous complexification of networks arranged in nested hierarchies. This by no means suggests that one level of aggregated complexity is more difficult to deal with than a less aggregated one. In fact, the opposite is often true. For example, in the study of value-creating cyber–physical systems, the study of shop-floor management has been done for decades with almost solely qualitative methods and common sense [21,22,23]. Deep learning has been recently used to extract statistical patterns from cyber–physical systems at certain microscopic local levels [24,25]; however, there is an urgent need for algorithms to be developed that ensure a holistic understanding of cyber–physical systems at the meso- and macroscopic level of complex-networked aggregation.

Subsequently, a research hypothesis can be formulated. Due to the high potential shown by deep learning in a wide range of applications, we could hypothesise that deep learning can be used to find patterns within Industry 4.0 lean-management complex-networked cyber–physical systems, which takes us to the concept of geometric deep lean learning. The analogy of networks proposed in this work, as well as the global analysis of the evolving networks and, through the geometric deep lean learning of the local relations between agents, provide an adequate context to establish which data to collect, and how to structure their analysis in a general and systematic way.

Within this context, there are two main resource-organizing classes for integrating deep learning in Industry 4.0 cyber–physical contexts with regard to different assumptions on data acquisition:

- Offline training, and decision-support learning and predicting from a global and integrated way, for example, by extracting relevant information from an organization by means of deep-learning algorithms that analyze previously labeled text in organizational categories [66]. Alternatively, by combining deep learning with other computing methods that allow for more balanced datasets and, hence, better deep-learning performance [67].
- Digital twin and augmented reality. Creating virtual environments that, by recording, visualization, and interaction with cyber–physical assets, are capable of generating necessary tagged information in real time that is fed to deep-learning algorithms [68]. The creation of digital twins in combination with deep-learning algorithms was also proposed to enable the parallel control of cyber–physical value-creating processes [69].

Deep-learning algorithms are built by stacking data-processing filters—layers—in deep architectures [5]. These layers extract increasingly accurate representations of the data fed into them through a series of algebraic operations, such as convolution (learning local patterns of feature maps) and pooling (downsampling of feature maps). A key reason for the success of these classical deep-learning applications on time-series, images, or video processing, is on its underlying Euclidean or gridlike data-structure space. The ability to leverage statistical properties of such data through local statistics is possible because of the shift invariance, local connectivity and the multi-resolution of the dataset. For instance, in a color image, pixels are placed together (shift invariance), present local properties (local connectivity), and present a red–green–blue-layered color structure (multiresolution). The use of convolution and pooling imposes conditions on the dataset while extracting local features shared throughout images that make it suitable for the problem without sacrificing the expressive capacity of the network. In fact, the graph’s Laplacian L=D−A that supports the information contained in the images is constant [70], where *D* and *A* represent the degree and adjacency matrix of the graph, respectively [19]. This allows a series of mass algebraic operations that make the magic of deep learning possible. However, at an organizational level, networks associated with Industry 4.0 lean-management cyber–physical systems are, by definition, dynamic and do not present these characteristics.

The fundamental idea of deep learning is that it is assumed that data to be studied came from the combination of different attributes at multiple hierarchical levels. An important underlining concept in this context is that of the manifold. A manifold can be intuitively understood as locally Euclidean space. Earth, for example, can be understood as a gigantic ellipsoid, but to a human at a point on its surface, it appears to be a plane. In other words, the manifold is an interconnected region: a series of points associated with its surrounding environment. From any of these points, the manifold appears to be locally Euclidean. Formally speaking, differentiable *X* manifold of dimension *d* is a topological space in which each point *x* has an environment that is homeomorphic to a Euclidean space of dimension *d* called tangent space $T_{x}X$ [71]. If the manifold is equipped with a Riemannian metric, such as an inner product ${\langle \;\cdot \;,\;\cdot \;\rangle }_{T_{x}X}:\left(T_{x}X\right)x\left(T_{x}X\right)\to R$, then the manifold is called a Riemannian manifold. The set of tangent spaces at all points is known as tangent bundle TX and is assumed to be smoothly dependent on the *x* position. It is precisely this feature that is exploited by machine-learning algorithms. The condition for this is the implicit assumption that interesting points occur only in a collection of manifolds in directions tangent to the TX planes, and with statistically interesting variations happening only when switching manifolds.

In other words, manifolds are topological spaces locally homeomorphic to Euclidean spaces. Complex networks, the object of this study, can be described by complexes of nodes and edges (i.e., triangles) that can be treated as discrete types of manifolds [72]. As has been described before [73,74,75], these can be understood as manifolds in order to explain the problems related to evolutionary manifolds using the theory of complex evolutionary networks. Specifically, deep learning applied to graphs usually considers these as manifolds; for this reason, we can consider deep lean learning as a manifold learning challenge. In the following sections, the consideration of graphs as manifolds is not geometrically rigorous, and might not be as smooth as previously defined. Classical applications of deep learning to graphs [76] focuses on static networks, but cyber–physical systems represented by complex networks are dynamic in nature, as nodes (both human and cyber–physical) and sociotechnical relations between them are constantly evolving.

For this reason, in order to discover statistical patterns within lean-management cyber–physical systems by means of deep learning, it is necessary to either transform existing data into figures that can be interpreted by classical approaches, or to generalize the concept of deep learning to dynamic networks. The first strategy was successfully implemented by one of the authors [13]. The second strategy follows in the footsteps of geometric deep learning.

Geometric deep learning is an emerging technique to generalize deep-learning models to non-Euclidean domains, such as certain graphs and manifolds [70]. The wide variety of domains in which geometric deep learning has so far been useful can be summarized in four categories:

- Graphwise classification. For instance, in the classification of molecules [77]. In this model, atoms represent the nodes, and chemical bonds are the edges of a graph. Research aims to extract certain features that predict certain properties of the molecule. This is relevant, for instance, to the pharmaceutical companies that are in the business of drug design. Some of these properties are toxicity and water solubility. Given a graph, researchers aim to classify a molecule graph. This is analogous to classical deep-learning-based visual image classification [78].
- Vertexwise classification. For example, in a social-network domain in which nodes are people of which we have certain demographic information, a researcher aims to predict how these people will vote in the next election. The analogy in computer vision is semantic image segmentation [79] in which the pixels of an image are labeled as belonging to a certain category.
- Graph dynamics. There are also domains that are described by fixed graphs, and others in which the graph changes with time [70]. Complex-networked cyber–physical systems belong to the second class.
- Known vs. unknown domain. In some cases, the graph can be known; in others, it is only partially known, *noisy*, or not known at all and needs to be learned. In these cases, the researcher aims to not only learn the graph features, but also the graph itself [80].

Existing approaches to implement geometric deep learning can be classified into two broad categories: spectral and local filtering methods.

- Spectral filtering methods.
  Spectral filtering methods make use of the spectral eigendecomposition of the Laplacian graph to elegantly mathematically define convolution-like operators. The fundamental limitation of the spectral construction is that it can only be used to single and static domains. This is because filter coefficients are dependent on the eigenvector- and eigenvalue-decomposition basis of the Laplacian graph, which is highly dependent on network architecture [70]. This approach is not suitable for our needs because of the dynamic characteristics of Industry 4.0 lean-management cyber–physical complex systems and their associated complex networks.
- Local filtering methods.
  Local filtering methods, on the other hand, are not topology-dependent, fall within the frame of signalling processing on graphs [81], and are more suitable in this setting, in particular, in order to define an operation similar to convolution in this domain [82].

## 4. Geometric Deep Lean Learning Over Industry 4.0 Lean-Management Complex-Networked Cyber–Physical Systems

According to Immanuel Kant, a science is not a science until there is a relation to mathematics. Although this characterization is provocative, and few would discuss such absolute numbers today, the implicit main question remains valid: can we find mathematical expressions that explain, process, and learn from network data, especially from complex-networked cyber–physical systems? This question is the motivator of this work, both for its practical and theoretical interest. On the one hand, empirically speaking, the processing of signals on graphs from complex cyber–physical networks has exponential importance due to the unstoppable emergence of technologies such as the IIoT and blockchain. On the other hand, the theoretical field of artificial intelligence constantly needs to develop new algorithms and computational architectures to later allow its practical application.

Applied to the analysis of complex-networked cyber–physical systems in the context of Industry 4.0, this leads to two classes of problem formulations that geometric deep lean learning theoretically solves:

- Strategic organizational design. Performing classical inference problems [76].
  Recently, it has been shown that this classification can be considerably improved by using information about the proximity environment [83,84]. Analyzing signals on graph vertices and edges could potentially help to learn inherent structures of the graphs, such as organizational clusters, with better accuracy than that provided by topological information alone—this is a strategic challenge to which organizational design tries to respond.
- Trust and power structures. Learning hidden organizational properties.
  Although deep learning has been employed in a wide variety of fields of knowledge, such as modeling social influence [85] and computer vision [86,87,88], it is important to incorporate knowledge about the domain to be treated in the model. For example, in order to build a deep-learning model for the study of a network of sensors in a cyber–physical system of industry 4.0, it might be useful, in a first approximation, to choose the edge weights of the graph as a decreasing function of the distance between nodes, as this would lead to a smooth graph signal model [89]; however, this would not be suitable for a lean structural network, because adjacency does not necessarily mean similarity [14]. For this reason, the model of the graph to be used can be superimposed on other structures, instead of being a pure unconnected abstraction. In other words, the graph that represents the complex-networked cyber–physical system in an Industry 4.0 context, can be studied from different perspectives, superimposing it to a specific sociotechnical environment that helps to better understand the statistical information that it contains. As a consequence, the integration of these priors is a fundamental challenge for the success of geometric deep lean learning. Some examples are the structures of power or trust between the different actors of an organization that are fundamental variables that influence the success of an organization, but remain elusive, since they often cannot be directly measured. Geometric deep lean learning could be applied to learn these parameters as weights between the nodes of the complex organizational network.

These problems reduce to fitting a time-dependent tensor A(t), so that Ω(t+1)≈A(t)·Ω(t) [90]. The hypothesis underlying this objective is that x(t+1)≈A(t)·x(t) where A(t) is constant in a window of time. The reason why we can take this assumption as true is that complex networks associated with cyber–physical systems in Industry 4.0 environments do not have very high variability [14]. As a result, a sufficiently small time window can always be found in which the hypothesis is sufficiently true.

**Figure 2:**
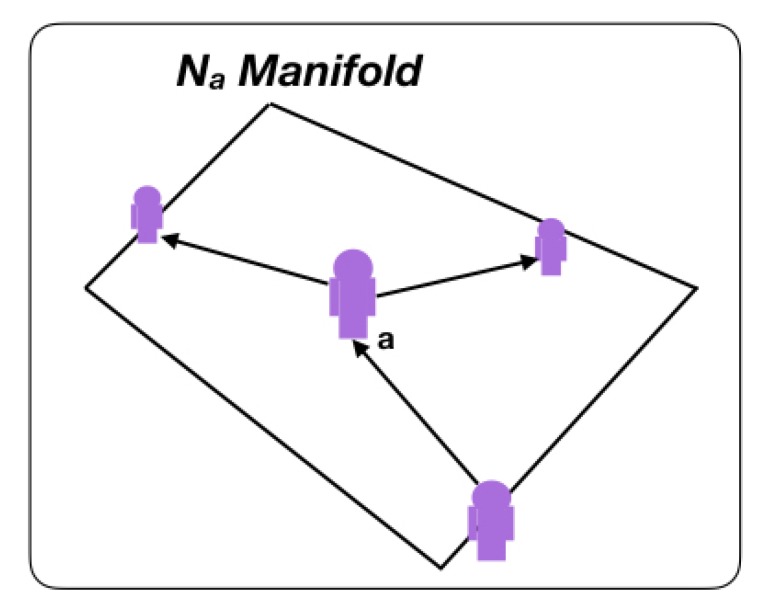
Local manifold upon which graph convolution acts.

Generalizing deep-lean-learning models to dynamic structured data in complex graphs requires a detailed description of the non-Euclidean equivalents to the basic elements of deep learning (convolutional layers and downsampling “pooling”), locally applied to each of the graph elements [70]:

- Convolution on non-Euclidean complex-networked cyber–physical graph time-dependent signals.
  As expressed in Expression 1, for weighted time-dependent directed graph Ω(t), a series of signals $x=\left[x\left(1\right),\ldots ,x\left(n\right)\right]\in \left(R^{n}xR^{m}\right)$ expressed on its human and cyber–physical nodes, and on its standard communication edges, are considered, in which components of $x_{a}$ reside in or are protruding from node *a*.
  For each node, we define a proximity environment given by group $N_{a}=\left\{b:\left(b,a\right)\subset E\right\}$ that represents set of nodes *b* connected with *a*. This $N_{a}$ set is characterized by an $R^{NxN}$ matrix *S* called the network-translation matrix operator that defines the manifold metric. We defined *S* as the graph adjacency matrix, the Laplacian of the graph, or any other normalization of it, as a linear transformation to encode the structure of a graph. Without loss of generality, the singularity problem of the adjacency matrix, which is nontrivial, was not considered in this work [91]. As shown in Figure 2, group $N_{a}$ represents the manifold upon which the convolution acts.
  The Fourier decomposition of graph Ω(t) is expressed by $\hat{x}=U^{-1}\;\cdot \;x$, where $S=U\;\cdot \;\Lambda \;\cdot \;U^{-1}$ and autovalues Λ describe the frequencies of the graph [92]. Now, we can directly filter *x* from the spectral domain by means of function f:C→R that allows to compute convolution $\hat{z}=f\left(\Lambda \right)\;\cdot \;\hat{x}$ by means of point-by-point multiplication in the spectral domain between filter f(Λ) and the Fourier transform of the graph in *x*. Therefore, by inverting the Fourier transform of the graph, we obtain the extension of the convolutional operation to the non-Euclidean time-dependent graph in Equation (2).
  (2) $$\begin{array}{c} z=P\left(S\right)x\;\mathrm{and}\;P\left(S\right)=Uf\left(\Lambda \right)U^{-1} \end{array}$$
  The filter operation can be directly described on the node, resulting in an alternative formulation given by Equation (3), where scalar parameter $\phi_{a,b}$ is a representation of the information weights coming from neighbour node *b* into or from node *a*.
  (3) $$\begin{array}{c} z_{a}=\sum_{b\in N_{a}\cup a}^{}\phi_{a,b}\;\cdot \;x_{b} \end{array}$$
  Due to the local properties of *S*, $z_{a}$ can be obtained in the domain of the node through local-information exchange. This means that the initial signal on the node is recursively transformed by *S* a *K* number of times until decomposition is obtained that determines $z_{a}$ as the convolution between the network filter with a polynomial transfer function and $x_{b}$.
  By means of the Fourier transform of the network, the screening operation of Equation (3) has the transfer function given by Equation (4):
  (4) $$\begin{array}{c} h\left(\Lambda \right)=\sum_{k=0}^{\kappa }\phi_{k}\;\cdot \;\Lambda^{k} \end{array}$$
  This filter, based on local-information exchanges, captures information in K-radius proximity from the node representing the depth of the geometric-deep-lean-learning algorithm.
  Taking into account this convolutional operation given by Equation (3), we are able to compute the *f*th level feature produced as output of the *l*th layer:
  (5) $$\begin{array}{c} y_{f}^{l}=\sigma^{l}\;\cdot \;\left(\sum_{g=1}^{l-1}P_{f,g}^{l}\;\cdot \;y_{g}^{l-1}\right) \end{array}$$
  where:
  - $\sigma^{l}$ represents the nonlinear activation function (i.e., ReLU); and
  - $P_{f,g}^{l}\;\cdot \;y_{g}^{l-1}$ indicates the graph structure relating the *g*th input $y_{g}^{l-1}$ to the *f*th output $y_{f}^{l}$.
  Now, we simply combine two cases to model the mechanism of a convolutional network applied to a non-Euclidean graph in each time slot: the case in which edges vary, and that in which nodes vary. This can be combined into a single expression to describe $P_{f,g}^{l}$ given by Equation (5):
  (6) $$\begin{array}{c} P_{f,g}^{l}\left(S\right)=\sum_{k=1}^{K}\Upsilon_{f,g}^{l,(k:1)}+\sum_{k=0}^{K}\left(\prod_{m=0}^{k}\Upsilon_{d}^{\left(m\right)}+\phi_{k}\;\cdot \;\tau^{k}\right) \end{array}$$
  where:
  - $\sum_{k=1}^{K}\Upsilon_{f,g}^{l,(k:1)}$ represents the edge-varying case, in which
    * $\Upsilon_{f,g}^{l,(k:1)}$ acts as a shift operator, and therefore represents a learning paradigm for data embedded within complex graphs, whose weights are known to some degree of ambiguity, are only partially known, or are unknown.
  - $\sum_{k=0}^{K}\left(\prod_{m=0}^{k}\Upsilon_{d}^{\left(m\right)}+\phi_{k}\;\cdot \;\tau^{k}\right)$ represents the node-varying case, in which
    * d⊂E is a special set of nodes (i.e., nodes with a degree above a certain threshold, nodes with a certain level of hierarchy in the organization, or any other relevant feature),
    * $\phi_{k}\in {\left[0,1\right]}^{Nxd}$ is a binary matrix, and
    * $\tau^{k}$ is a vector describing the node parameters in *d*.
- Pooling in non-Euclidean complex-networked cyber–physical graph time-dependent signals.
  As introduced earlier, downsampling pooling layers in classical deep-learning architectures that extract information from Euclidean domains such as speech, images, or videos typically report the maximal output within rectangular proximity [93]. In this way, it is possible to extract local characteristics that are shared by other areas of the images, thus considerably reducing the number of parameters that the deep network has to learn without sacrificing its learning capacity. Pooling can be described as a progressive coarsening of the graph. A simple way to do this is to collapse edges and reduce the size of the graph through a standard max-pooling operation on the nodes by just taking the maximum of each one of the feature tensors on each of the nodes being coarsened. This can be represented as a binary-tree structure of node indices. These pooling modules on graphs can be inserted between the convolutional modules in order to extract high-level graph representations, and thus be able to perform effective graph classification.
  Some alternatives in this field have not been to try to pool the whole network, but different hierarchies of the complex network in order to be able to learn which node groups have similar characteristics [94]. Once these groups are learned, clusters are made, and network pooling is carried out as described above or with an alternative method. This process is repeated for each of the network layers; thus, its classification is obtained. This presupposes, however, prior knowledge of the network structure.
  The extraction of shared local characteristics is not possible through this method in time-varying non-Euclidean domains, i.e., complex-networked cyber–physical graphs, because no stationarity or shift invariance can be found within these domains. Wu et al. [95], and Lee et al. [96] provided state-of-the-art surveying overview of this interesting open research question.

## 5. Conclusions and Management Implications

Geometric deep lean learning at a strategic level is expected to ensure sustainable organizational growth because customers and suppliers are able to reach new levels of transparency and traceability on the quality and efficiency of the processes, which generates new business opportunities for both, and new products, services, and co-operation opportunities in a cyber–physical environment. In a world of limited resources, increasing business volume can only be achieved by increasing the depth of integrated intelligence capable of successfully handling the emerging complexity in value streams. The future implications of geometric deep lean learning at an organizational level are yet to be fully deployed, but it is expected that the field of analysis of complex-networked cyber–physical systems in Industry 4.0 environments will attract intense attention from both industry and scholars who could develop tools to interpret, classify, and better understand the behavioral patterns of such networks through the application of this very exciting field of artificial intelligence.

Managerial implications of geometric deep lean learning on a mesoscopic level should try to integrate geometric deep lean learning in whole-value-stream processes to substantially improve resource optimization. Geometric deep lean learning at a value-stream level is expected to impact lead time and on-time delivery. At a mesoscopic level, producing only what the customer needs, when they needs it, in the required quality, the integration of deep-learning technologies is expected to not only allow the systematic improvement of complex value chains, but also the better use and exploitation of resources, thus reducing the environmental impact of Industry 4.0 processes. This technology could also be implemented at the customer side to increase defect-detection accuracy on products themselves. Such analyses provide sensitivity about operations and operational conditions, which also impacts value-stream-related efficiency and effectiveness.

The theoretical implications of the application of these geometric-deep-lean-learning models to data embedded within complex-networked datasets support researchers in departing from “crafted” features in modeling machine-learning models when dealing with geometric data. In the context of Industry 4.0 cyber–physical systems, these could be drone-positioning and decision-making algorithms, and the proper interpretation of wearable devices (i.e., physical sensors) on human or cybernetic process owners. Until now, models dealing with such problems required a certain amount of prior knowledge (e.g., the isometric-shape-deformation model), and often did not capture the full complexity and wealth of data. Geometric-deep-lean-learning methodologies could bring a breakthrough to the field and be the first indications of a coming paradigm shift by, for instance, expanding existing social-manufacturing knowledge into unknown territory through the contextual self-organizing of mass-individualization processes under a social-manufacturing paradigm through a cyber–physical–social system approach.

Some of the main potential applications can be clustered four categories:

- Graphwise classification. The classification of complex cyber–physical graphs by deep lean learning, thus creating product families and allowing automated decision making in real time in which products are developed, produced, and channeled to the final customer.
- Vertexwise classification. The classification of certain crucial nodes in the value-creation process by means of deep-lean-learning models that allows an improvement of organizational design to assure an increase of overall process performance.
- Graph dynamics. Learning complex-networked cyber–physical graph dynamics is of great interest when dealing with change management within non-Euclidean sociotechnical systems.
- Known vs. unknown domains. The learning, generation, and semisupervised design of value streams by learning the most suitable complex cyber–physical graphs for certain types of products, thus potentially generating high customization with high efficiency and effectiveness in resource use.

The data needed to implement these mathematical concepts are enormous and fall within the field of big data. The acquisition of data associated with the cyber–physical systems of Industry 4.0 is costly and of great strategic value to the involved organizations, which is why systems that increase the confidence of the involved actors and guarantee the security of these IIoT data, as the distributed ledger technology, are essential for the practical application of the exposed concepts. The quality of the obtained data essentially depends on the trust that the various value-creating actors have in each other. Achieving the necessary high degrees of confidence and successfully managing these parameters in an environment of interdependent supplier and customer networks is one of the challenges in the immediate future, and ought to be met by several blockchain and distributed ledger protocols. The Constrained Application Protocol is excellent for use with limited devices and low-power networks, such as those preferred in IIoT. To ensure greater security, applications known by the more important User Datagram Protocol, such as Voice over IP/Session Initiation Protocol, Datagram Transport Layer Security, can be run on User Datagram Protocol instead of Transmission Control Protocol. The Rivest–Shamir–Adleman hybrid algorithm can also be good, with high efficiency, better security and privacy protection, and is suitable for the end-to-end encryption requirements of the future IIoT. Future IIoT research within an Industry 4.0 complex-networked cyber–physical context should focus on, among others, the following characteristics: the open security system, the way in which individual privacy is protected, terminal-security function, and laws related to IIoT security. It is undeniable that IIoT security requires a set of policies, laws, and regulations, and a perfect security-management system for mutual collocation to ensure the success of this exciting and fruitful research endeavor.

## Figures and Tables

**Table 1:** Research overview.

	Social	Technical	Socio Technical
Micro	Imai, 2012 [26]; Stock and Seliger, 2016 [27]	Takeda, 2009 [28]; Francis and Bian, 2019 [29]; Jabeur et al., 2015 [17]; Li et al., 2017 [30]; Aazam et al., 2018 [31]; Tao et al., 2018 [32]; Mushtaq and Haq, 2019 [33]; Shevchik et al., 2019 [34]; Al-Jaroodi and Mohamed, 2019 [35]; Sun et al., 2019 [36]	Villalba-Diez et al., 2015 [23]; Villalba-Diez et al., 2019 [24].
Meso	Rother, 2010 [37]; Villalba et al., 2018 [13]; Birkel et al., 2019 [38]	Takeda, 2011 [39]; Davis et al., 2012 [40]; Gomez et al., 2015 [41]; Culot, 2019 [42]; Jimenez et al., 2016 [43]; Wang et al., 2018 [44]; Villalba-Diez et al., 2019 [25]; Jang et al., 2019 [45]; Ordieres-Mere et al., 2019 [46]	Villalba-Diez and Ordieres-Mere, 2015 [10]; Villalba-Diez et al., 2015 [9]; Villalba-Diez and Ordieres-Mere, 2016 [11]; Villalba-Diez et al., 2017 [47]; Davies et al., 2017 [48]; Kumar et al., 2019 [49].
Macro	Womack and Jones, 2003 [50]; Toyota, 2014 [51]; Burton et al., 2015 [52]; Covey, 2004 [53]; Rabelo et al., 2019 [54]; Romero et al., 2017 [55]; Wang et al., 2019 [56]; Guo and Jyang, 2019 [57]	Lee et al., 2015 [58]; Wang et al., 2015 [59]; Goodfellow et al., 2016 [5]; Sisini et al., 2018 [60]; Zheng et al., 2018a [61]; Lu and Xu, 2019 [62]	Stock and Seliger, 2016 [27]; Villalba-Diez, 2017 [15]; Villalba-Diez, 2017 [14]; Kiel et al., 2017 [63]; Stock et al., 2016 [64]; Shang et al., 2019 [65].

## Abbreviations

The following abbreviations are used in this manuscript:

IIoT	Industrial Internet of Things
